# Face Value: Towards Robust Estimates of Snow Leopard Densities

**DOI:** 10.1371/journal.pone.0134815

**Published:** 2015-08-31

**Authors:** Justine S. Alexander, Arjun M. Gopalaswamy, Kun Shi, Philip Riordan

**Affiliations:** 1 The Wildlife Institute, School of Nature Conservation, Beijing Forestry University, Beijing, China; 2 Wildlife Conservation Research Unit, Recanati-Kaplan Centre, Department of Zoology, University of Oxford, Tubney, Abingdon, United Kingdom; 3 Department of Zoology, University of Oxford, Tinbergen Building, South Parks Road, Oxford, OX1 3PS, United Kingdom; 4 Wildlife Without Borders UK, Abingdon Road, Oxfordshire, OX13 5QL, United Kingdom; University of Lleida, SPAIN

## Abstract

When densities of large carnivores fall below certain thresholds, dramatic ecological effects can follow, leading to oversimplified ecosystems. Understanding the population status of such species remains a major challenge as they occur in low densities and their ranges are wide. This paper describes the use of non-invasive data collection techniques combined with recent spatial capture-recapture methods to estimate the density of snow leopards *Panthera uncia*. It also investigates the influence of environmental and human activity indicators on their spatial distribution. A total of 60 camera traps were systematically set up during a three-month period over a 480 km^2^ study area in Qilianshan National Nature Reserve, Gansu Province, China. We recorded 76 separate snow leopard captures over 2,906 trap-days, representing an average capture success of 2.62 captures/100 trap-days. We identified a total number of 20 unique individuals from photographs and estimated snow leopard density at 3.31 (SE = 1.01) individuals per 100 km^2^. Results of our simulation exercise indicate that our estimates from the Spatial Capture Recapture models were not optimal to respect to bias and precision (RMSEs for density parameters less or equal to 0.87). Our results underline the critical challenge in achieving sufficient sample sizes of snow leopard captures and recaptures. Possible performance improvements are discussed, principally by optimising effective camera capture and photographic data quality.

## Introduction

Many large carnivores are keystone species [1] whose population declines can lead to dramatic ecological effects when their densities are reduced below certain thresholds, leading to oversimplified ecosystems [2–4]. Their high metabolic demands [5] and prey requirements [6] make them highly prone to conflict with humans and livestock [7]. These threats leave large carnivores vulnerable to steep declines in numbers, with major ecological consequences [8]. However, we often lack information on the density and distribution of large carnivores because they occur in low densities, cover wide ranges and are often cryptic, making their density estimation a challenging endeavour [9].

The endangered snow leopard *Panthera uncia* [10] is one such large carnivore felid for which these concerns are accentuated. Estimating density reliably is particularly demanding because the snow leopard is found in largely inaccessible and difficult terrain, at high elevations [11]. In this study, we designed and developed a photographic spatial capture-recapture survey of snow leopards in China to estimate their density and to explore the influence of environmental and human activity indicators on their spatial distribution.

The global population of snow leopards is roughly estimated (as of 2003) at 4,000–6,500 individuals [12] based on calculations of habitat suitability. Threats to snow leopards vary across their range and views on their relative importance are largely based on expert opinion. Until recently, researchers primarily depended upon the detection of physical signs to verify the presence of snow leopards and assess their relative density or abundance across study sites [13–15]. New non-invasive techniques, such as the use of camera traps, are now available as powerful tools for developing density estimates for felids such as snow leopards [9,16]. Camera traps are suitable for individual identification of snow leopards using morphological characteristics (e.g., uniquely identifiable coat patterns)[11].

Capture-Recapture (CR) methods are one of the primary means of assessing carnivore population numbers and dynamics [17]. However the estimation of population density using conventional closed CR models [18,19] is dependent on largely subjective assessment of the appropriate size of the effective sampled area. In that sense, CR models represent an *ad hoc* approach [20]. Spatially Explicit Capture Recapture (SECR) models incorporate spatial locations of captures within a unified model and provide more reliable estimates of density [17,21–23]. SECR models have been applied to variety of large carnivores, such as tigers *Panthera tigris* [22], black bears *Ursus americanus* [24], jaguars *Panthera onca* [25]. Up until now they have not been applied to snow leopards. These models have the potential not only to assess densities but also to quantify spatial distributions and associated threats. In the application of SECR models there is need for innovative approaches to gathering data from multiple sources and combining them to build a more complete picture of the population dynamics and ecological drivers.

A relatively small number of studies using camera trap data in the CR framework have been conducted on snow leopards to estimate their population abundance and/or density [11,15,26,27]. In those that carried out density estimates, conventional CR approaches combined with an *ad hoc* estimation of buffer width, and often small sample sizes (typically fewer than 15 individuals), give rise to concerns about their precision and reliability [11,15,28,29]. The use of such estimates based on low sample sizes should, therefore, be subject to caution [30], especially for rare carnivores such as snow leopards. As the costs of camera equipment have decreased, camera trap surveys are becoming much more common throughout the snow leopard range. This presents an opportunity to freshly examine current methodologies, in order to provide greater rigor in developing density estimates.

This paper describes the use of non-invasive data collection techniques combined with recent SECR methods to provide an estimate of snow leopard densities in North Central China. We also combine different survey techniques in order to examine spatial distribution and weigh ecological determinants.

We, thus, set the following specific objectives in our study:

1. To develop rapid and cost-effective camera trap survey methods to gather snow leopard photographic data amenable to SECR analysis in an understudied region in the snow leopard range, the Qilianshan National Nature Reserve (QNNR), Gansu Province, China.
2. To estimate snow leopard densities using Bayesian SECR approaches and assess the reliability of these density estimates using computer simulations based on our study design and camera trap-operating scheme.
3. To assess environmental and human factors that influence snow leopard density and distribution and make recommendations for future research and conservation practice.

## Materials and Methods

### Ethics statement

The study was conducted in the protected area of QNNR within the Province of Gansu, China. China’s State Forestry Administration reviewed all sampling procedures and approved permits for the work conducted. We applied non-invasive methods for data gathering and hence approval from an Institutional Animal Care and Use Committee or equivalent animal ethics committee was not required.

### Study area

The Qilianshan mountain range lies on the North-eastern margin of the Tibetan Plateau (38° N, 98° E). The QNNR was established in 1988 by China’s State Council to protect forest and wildlife and covers a total area of 26,530 km^2^ [31]. Habitat of the QNNR primarily consists of grassland (mainly *Stipa przewalskii* montane grassland and *Polygonum viviparum* alpine grassland) and shrub vegetation (*Caragana spp*.) with isolated areas of forest (*Picea crassifolia* coniferous and *Sabina przewalskii* forest) [32]. The QNNR area supports a mammalian carnivore assemblage, consisting of snow leopard, brown bear *Ursus arctos*, lynx *Lynx lynx*, grey wolf *Canis Lupus* and red fox *Vulpes vulpes*, as well as smaller species of felids and mustelid, while blue sheep *Pseudois nayaur* and white-lipped deer *Przewalskium albirostris* are the main wild ungulates within the area.

The assessment was carried out in the Northern region of QNNR, known as QiFeng (39.25° N, 98.74° E). Within the study area some livestock herding and leisure activities are permitted. However livestock herding is regulated and is limited to specific zones according to a policy issued in 2010 by China’s State Council, with the aim of re-generating overgrazed alpine vegetation.

### Data collection

**Fig 1:**
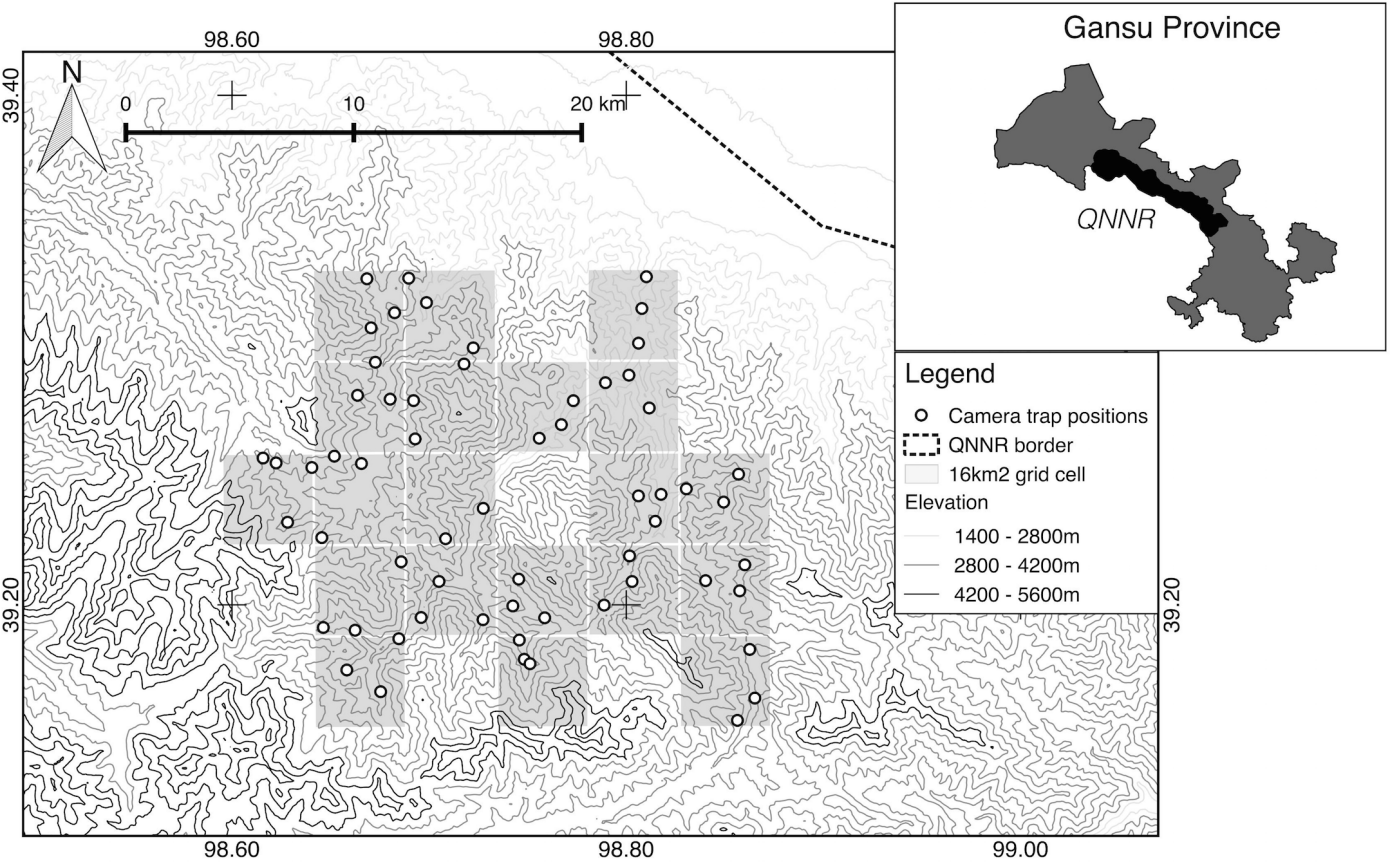
Study Area. Location of camera traps in QNNR, Gansu Province, China.

A total of 60 camera traps (RECONYX and Ltl Acorn) were systematically set up over the 480 km^2^ study area between January and March 2013 (Fig 1). This area size was considered adequate to capture multiple snow leopard home ranges as it is larger than published home range estimations ranging from 11–142 km^2^ [14,33]. The study area was divided into 20 grid cells of 4×4 km, ensuring even coverage of the whole area. Within all but two of the grid cells, three camera stations were set up, each consisting of a single camera unit [26]. Difficult terrain prevented more than two camera traps being placed in one of the grid cells, so four camera traps were placed within an adjacent cell.

Camera stations were placed with a minimum spacing of 1 km in order to simultaneously achieve the twin objectives of maximizing the number of individuals caught and adequately recapturing individuals at different camera traps, as required in SECR designs [21,22]. Three camera traps, however, were separated by distances of 310 m, 612 m and 821 m, respectively, due to topography.

The exact location of individual camera trap stations was chosen to maximize the possibility of detecting snow leopards, either at sites indicating the presence of snow leopard from signs, or at sites presenting natural pathways for snow leopards to walk through. We placed each camera trap station in such a way as to capture unique markings on the forehead of the animal, forgoing the traditional approach of placing a second camera trap to capture both sides of the animal [11]. This was a crucial trade-off, which we decided upon because snow leopard images do not exhibit obvious sexual differences. By allowing for this concession, we were able to double our survey effort (especially in terms of area) for the available camera trap resources.

We limited the sampling period to 3 months to respect the CR closure assumption [34]. Cameras were left active and undisturbed for between 10–87 days in order to maximize the number of snow leopards captured on camera.

### SECR data analyses

We estimated population densities of snow leopards using a Bayesian SECR model [22]. This model is implemented in the R package, SPACECAP [35] (version 1.1.0) [36] running in R (version 3.1.1)[37].

Capture incidences were reviewed independently by two separate observers to identify individual snow leopards, using the snow leopard’s coat patterns as unique identifiers. The observers then jointly reviewed any discrepancy in identification (11 capture events) and a final agreement was reached on the identity of twenty individuals and their capture history. The identification of individuals was limited to comparing patterns on the face, forelimbs or posterior body. Each individual snow leopard was given a unique identification number [38]. Each day was considered a unique sampling occasion. We then constructed individual spatial capture histories for all the 20 individuals photographed, irrespective of age class, on the 93 trap occasions in 60 traps using the procedure prescribed in SPACECAP [36]. The data were then formatted for SPACECAP analysis [35]. The potential home range centres (also known as state space) were generated for the effective trapping area, an area within 24 kilometres buffer distance surrounding the sampled area. Preliminary analyses indicated that density estimates stabilized prior to a 24 km buffer distance and that a further increase in buffer width did change density estimates. In our study, the state-space was described by equally spaced points in a regular grid, with a mesh size of 1.96 km^2^. This grid size was an optimum between being fine enough to minimize the error in estimating home range centres and large enough to reduce the overall computation time required. Home range centres falling over areas that did not present a suitable habitat for snow leopards were identified based on our field knowledge, were mapped using QGIS and Google Earth and were marked accordingly in the SPACECAP input file. Unsuitable habitat was considered as areas outside the nature reserve, villages, agricultural land, roads and development sites. SPACECAP was run using a half normal model, data augmentation increased to 200, with 60,000 iterations, a burn-in of 10,000, and a thinning rate of 1. In order to test other model types SPACECAP was again run using a negative exponential model and the non-spatial model. The movement of individuals between camera stations was investigated by calculating the maximum distance moved from the recaptured snow leopard individuals.

### Model simulation

Many factors affect the reliability of estimates from SECR models, including trap configuration [39], and the sensitivity of parameters in the SECR model [25]. Hence, it is difficult to conceive of all scenarios, for various species, scales and configurations, which could be affecting estimates. To ensure that we were obtaining reliable and precise estimates for our specific study, we used estimates from the final (half normal) model analysis to simulate 100 SECR data sets for the specific sampling situation used in our study. Each data set was run in SPACECAP using a half normal model; data augmentation increased to 200, with 30,000 iterations, a burn-in of 15,000, and a thinning rate of 1. This generated estimates of density and psi (the ratio of the estimated abundance within the state space to the maximum allowable number). We then evaluated these estimates using a frequentist statistical approach [40]. We computed root-mean-squared-error (RMSE) by averaging over the data-generating distribution, for the posterior mean, median and mode. We also calculated coverage rates for the 95% highest posterior density intervals.

### Identification of potential covariates

The density estimates from the SECR model were assessed against potential site covariates measured in our study. We used three continuous site covariates (prey presence, grazing activity and slope), which were hypothesized to influence the likelihood of habitat use of snow leopards (Table 1). These hypotheses were based upon responses from key informants at the local level.

**Table 1:** Factors hypothesized to influence patterns of snow leopard density in QNNR, with the corresponding index used, predicted direction of effect, source of data, and range of values across sampled state space (480 km^2^).

Factor	Index	Predicted effect	Source	Range of values
Prey presence	Relative proportion of blue sheep signs	Positive	Field Collected	0–9 index
Grazing activity	Relative proportion of livestock signs	Negative	Field collected	0–9 index
Slope	Slope Variance	Positive	SRTM 90 m digital elevation database V 4.1	6–13.5 standard dev.

The availability of suitable prey species is known to be a key determinant of the distribution and abundance of carnivore populations [6]. Snow leopards are opportunistic predators that exploit a wide range of prey species, with a predilection for large ungulates [41,42]. We used data collected in the field to create an index of abundance characterizing spatial patterns of the primary ungulate within the region, the blue sheep. Within each of the twenty 16 km^2^ grid cells, we completed between 3–9 km transects of sign surveys. Each 1 km transect was divided into four 250 m segments and all recent signs (tracks and remains of carcasses) of blue sheep presence were sought in every segment. We noted only tracks that were easily recognizable (sharply defined) and unambiguously identified. Additional blue sheep signs in the same 250 m segment were not recorded separately. We created an index of prey presence defined as the proportion of 250 m segments in each grid cell with the presence of blue sheep.

We also hypothesized that snow leopards would tend to avoid areas of human activity, represented in this study by livestock grazing [43]. Livestock grazing (primarily yak and small stock such as sheep and goats) is the main local livelihood and land use activity within the region [44]. In each 250 m transect segment, information was also collected on detection/non-detection of the presence of recent livestock grazing activity. Our index of livestock activity was the proportion of 250 m segments in which grazing was recorded.

We applied ordinary kriging [45–47], with log transformation to estimate and interpolate frequency of prey and grazing activity in non-sampled areas. This was done using the Geostatistical Wizard procedure within the Geostatistic analyst tool of ArcGIS 9.3 [48]. Spatial kriging generated a landscape level map of distribution probabilities for prey and grazing for our entire 480 km^2^ study site (S1 Appendix). Both these covariate layers were converted to a 1.96 km^2^ resolution using QGIS.

We also hypothesized that habitat heterogeneity would have a less pronounced effect on snow leopard occurrence patterns. Nevertheless, we predicted that at the local level, snow leopards would select particular areas for use and that their presence would increase in barren/broken terrain, steeper areas, and sparse vegetation [14,43]. Elevation data was obtained from SRTM 90m digital elevation database V 4.1. QGIS Version 2.2 Valmiera [49] was used to calculate the standard deviation of slope at the 1.96 km^2^ resolution. High values of the slope standard deviation variable indicate steep or irregular terrain. All three covariates (prey presence, livestock activity and slope standard deviation) were standardized and centred prior to analysis.

We were interested to explore the effect of selected covariates on snow leopard density in our study area. Covariate information was available to us for M = 252 cells in the study area. SPACECAP [35] version 1.1.0 [36] provides users with estimates of pixel-specific densities as one of its outputs. An obvious choice for exploring the influence of covariates is to use a homoscedastic model and regress pixel-specific snow leopard densities obtained from SPACECAP with corresponding pixel-specific covariates. However, since pixel-specific snow leopard density is essentially an outcome of a count variable in SPACECAP, it is more appropriate to use a generalized linear model [50] to model the influence of covariates.

**Fig 2:**
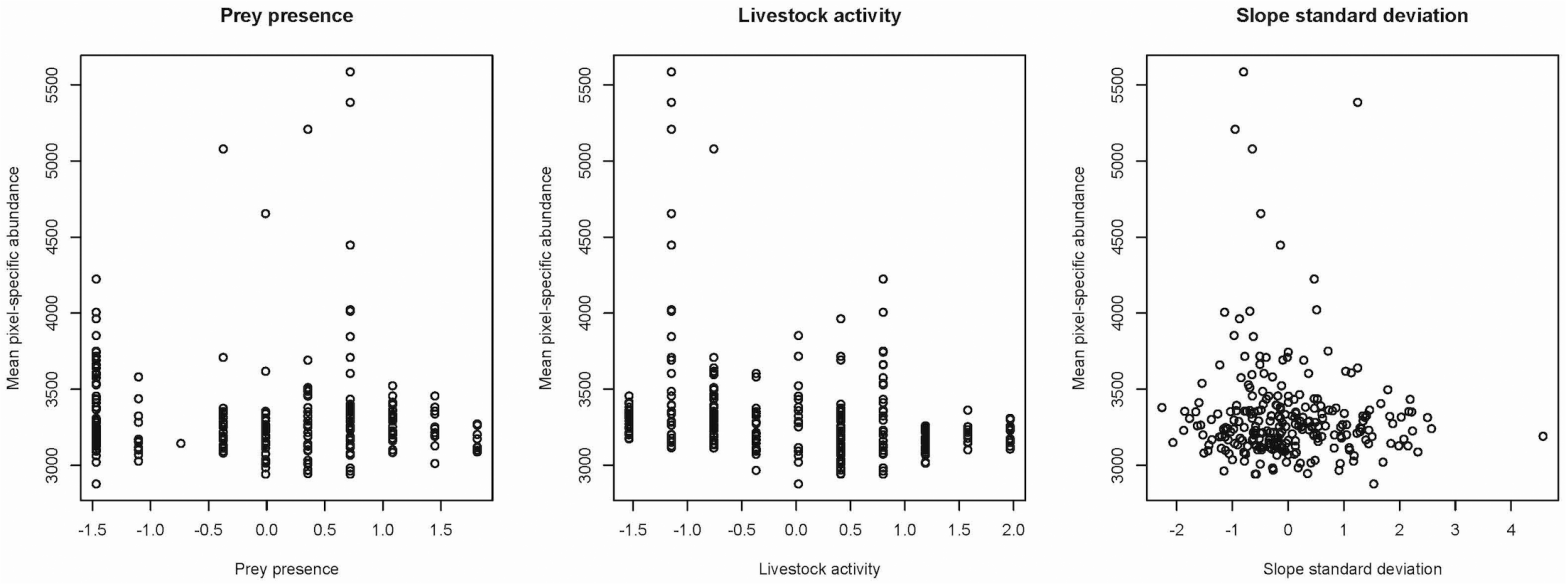
The mean pixel-specific abundance plotted against standardized covariates.

Each iteration in the MCMC analysis in SPACECAP provides a pixel-specific count vector of the number of snow leopards. We assumed that these counts on each pixel emerge from a count process such as the Poisson distribution or a Negative Binomial distribution to handle for over dispersion. We therefore took the mean of all the iterations in order to generate an estimate of mean snow leopard abundance for each pixel. When the mean pixel specific snow leopard abundance was plotted against each of the explanatory variables (Fig 2), it appeared that there was more over dispersion than was described by the simple Poisson process, where the mean is equal to the variance. We therefore utilized a Negative Binomial regression [51] to explore the effect of covariates on local snow leopard abundance.

We then implemented the Negative Binomial regression using the ‘glm.nb’ function in the ‘MASS’ package available in R [37]. An Information Theoretic (IT) approach [52] was used to explore the most appropriate Negative Binomial regression model and assess which covariates had greatest influence on snow leopard abundance[53]. The combination of all predictor covariates (prey presence, livestock activity and slope standard deviation) produced 7 separate Negative Binomial models. Akaike’s Information Criterion (AIC) was used to rank models. The relative importance of different parameters was assessed by summing AIC weights. The highest ranked models (all within 2.0 delta-AIC of the top model) were used to determine effect size and direction of beta coefficients [53]. All models were run in R 3.1.1[37].

We tested for goodness-of-fit the overall Negative Binomial regression model with a chi-square test based on the residual deviance and degrees of freedom. We directly compare the deviance residuals to the central Chi-squared distribution with N-p degrees of freedom, where p is the number of parameters. We considered a goodness-of-fit p value greater then 0.05 as indicative of an acceptable fit.

## Results

### Capture success

We recorded 81 snow leopard captures. We discarded 5 of these because either the same individual was captured by the same camera trap within a few hours’ interval (3 captures) or the captures were made within an incomplete twenty-four period (i.e. shortly before camera trap pickup; 2 captures). We therefore recorded 76 captures over 2,906 trap-days representing an average capture success of 2.62 captures/100 trap-days. A total of 30 camera traps (50%) captured snow leopards in 19 grid cells. On average, we obtained 1.26 (SE = 2.01, range = 0–11) captures/trap. Single snow leopards were mostly photographed, however a group of three individuals, possibly one female with two sub-adults, were captured on three occasions.

### Individual identification of snow leopards

**Fig 3:**
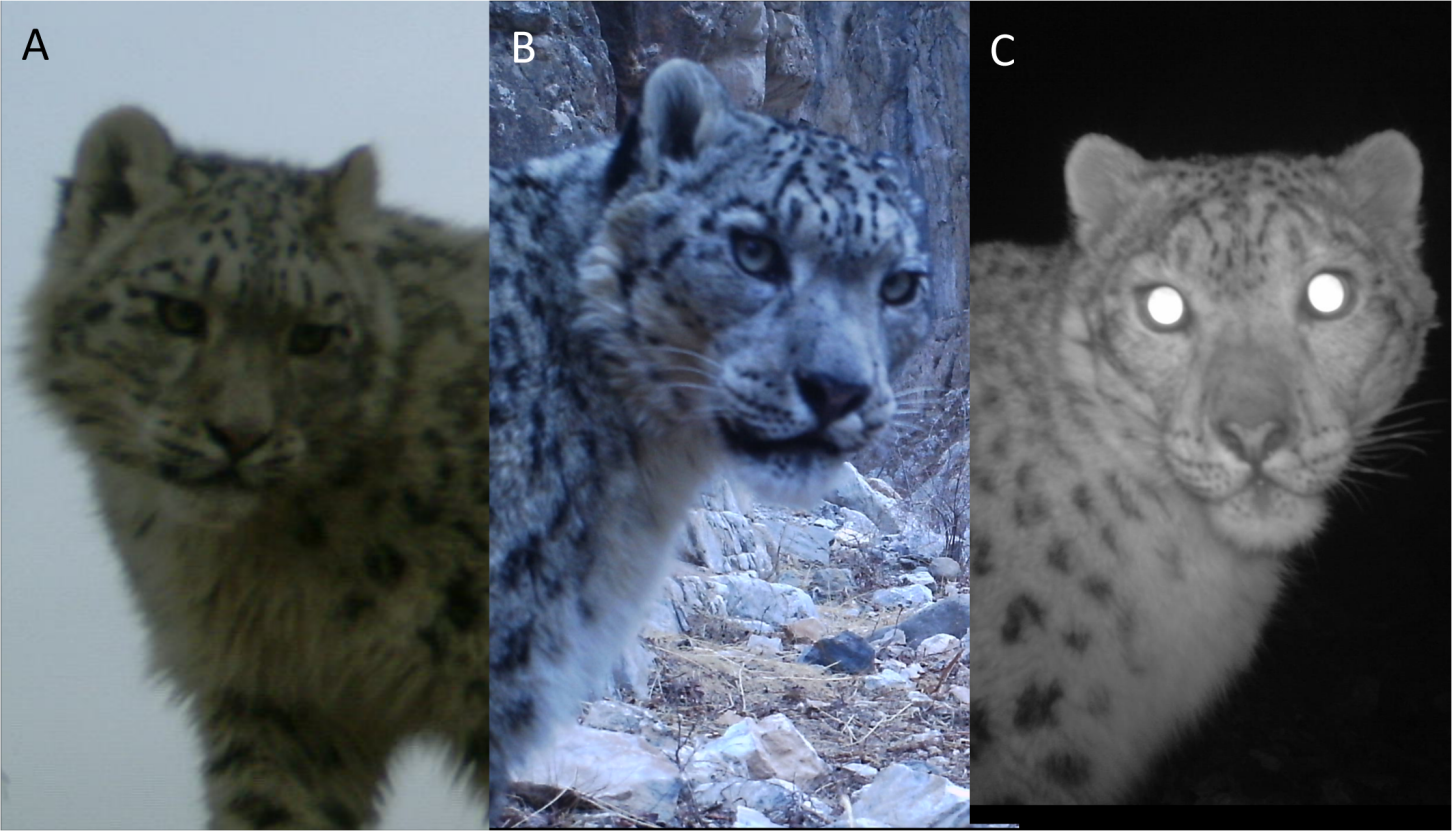
Snow leopard individual identification. B and C are photos of the same individual from different camera traps with C taken at night with infrared. A is a photo of a different individual. Identification is based on distinct spot patterns on the face.

Camera traps were set up to photograph individual snow leopards passing by in order to capture frontal features. Consequently most photographs showed the snow leopards head, with 25 (32%) of capture incidences (Fig 3). We photographed frontal limbs, left flank of torso, right flank of torso and tail in 23%, 23%, 21% and 23% of capture incidences. The most suitable body parts for identification were the facial features and the lower forelimbs. Inadequate lighting in night-time captures of the snow leopard individuals was the main cause of low quality images. Thirty-eight (50%) capture incidences were of insufficient quality to allow individual recognition. The remaining 38 (50%) captures were suitable for further analyses.

Using photos of sufficient quality, 20 individual snow leopards were identified for which capture histories were constructed. Twelve identified individuals were only captured once, four were captured twice, one three times, two four times, and one seven times. The number of individuals started to become asymptotic on the 78th day of 93 days of sampling. The 20 individual snow leopards captured included a group of three individuals of which two were assumed to be sub adults. These three individuals were considered as separate individuals in the analysis. The average maximum distance moved (MMDM) from the 8 recaptured individuals (capture > 1) from anywhere in the study was estimated at 7.60 km (SE = 4.16 km) and half MMDM estimated at 3.80 km (SE = 2.10 km).

### Density estimates

Posterior SECR summaries from fitting the model with the half-normal detection function are given in Table 2. With the original number of 20 identified individuals, the mean estimate of snow leopard density for the identified suitable habitat within the 24 km buffer zone is 3.31/100 km^2^ (with a 95% interval of 1.42–5.32). The non-spatial model reported an estimated density of 8.31/100 km^2^ (with a 95% interval of 6.05–10.90).

**Table 2:** Posterior summaries from Bayesian spatially explicit capture-recapture (SECR) of the model parameters implemented in SPACECAP. (Density is presented per 100 km^2^).

Parameter	Posterior Mean	Posterior SD	95% Lower HPD	95% Upper HPD
**Half normal model**				
density	3.3147	1.0093	1.4206	5.3197
sigma	4784.7201	1035.1265	3023.8348	6768.9534
lam0	0.0036	0.0012	0.0015	0.0060
beta	0.0000	0.0000	0.0000	0.0000
psi	0.4986	0.1539	0.2108	0.8087
N	109.6659	33.3936	46.0000	175.0000
**Exponential model**				
density	3.5122	1.0090	1.5717	5.4708
sigma	4138.5436	502.1055	3265.2193	5131.2889
lam0	0.0127	0.0053	0.0042	0.0231
beta	0.0000	0.0000	0.0000	0.0000
psi	0.5279	0.1537	0.2419	0.8379
N	116.2005	33.3830	52.0000	181.0000
**Non-spatial model**				
density	8.3100	1.3980	6.0500	10.9000
sigma	NA	NA	NA	NA
lam0	0.0005	0.0001	0.0003	0.0007
beta	0.0000	0.0000	0.0000	0.0000
psi	0.1280	0.0304	0.0735	0.1890
N	27.5000	4.6263	20.0000	36.0000

The estimate and precision produced in the half normal model were consistent with those for the negative exponential model, where estimated density was 3.51/100 km^2^ (with a 95% interval of 1.57–5.47). The Bayesian P-values of both our models ranged from 0.64 to 0.71, indicating that the models were of an adequate fit. The Geweke test for the half-normal model indicated that all model parameters converged with *z* scores falling between 1.64 and -1.64 (sigma = 0.62; lam0 = -1.41; psi = -0.49; N = -0.45). The Geweke test results for the negative exponential model, however, implied lack of convergence for at least one parameter (sigma = -2.05; lam0 = 1.21; psi = 1.36; N = 1.50). The half-normal model was therefore used in further analyses.

**Fig 4:**
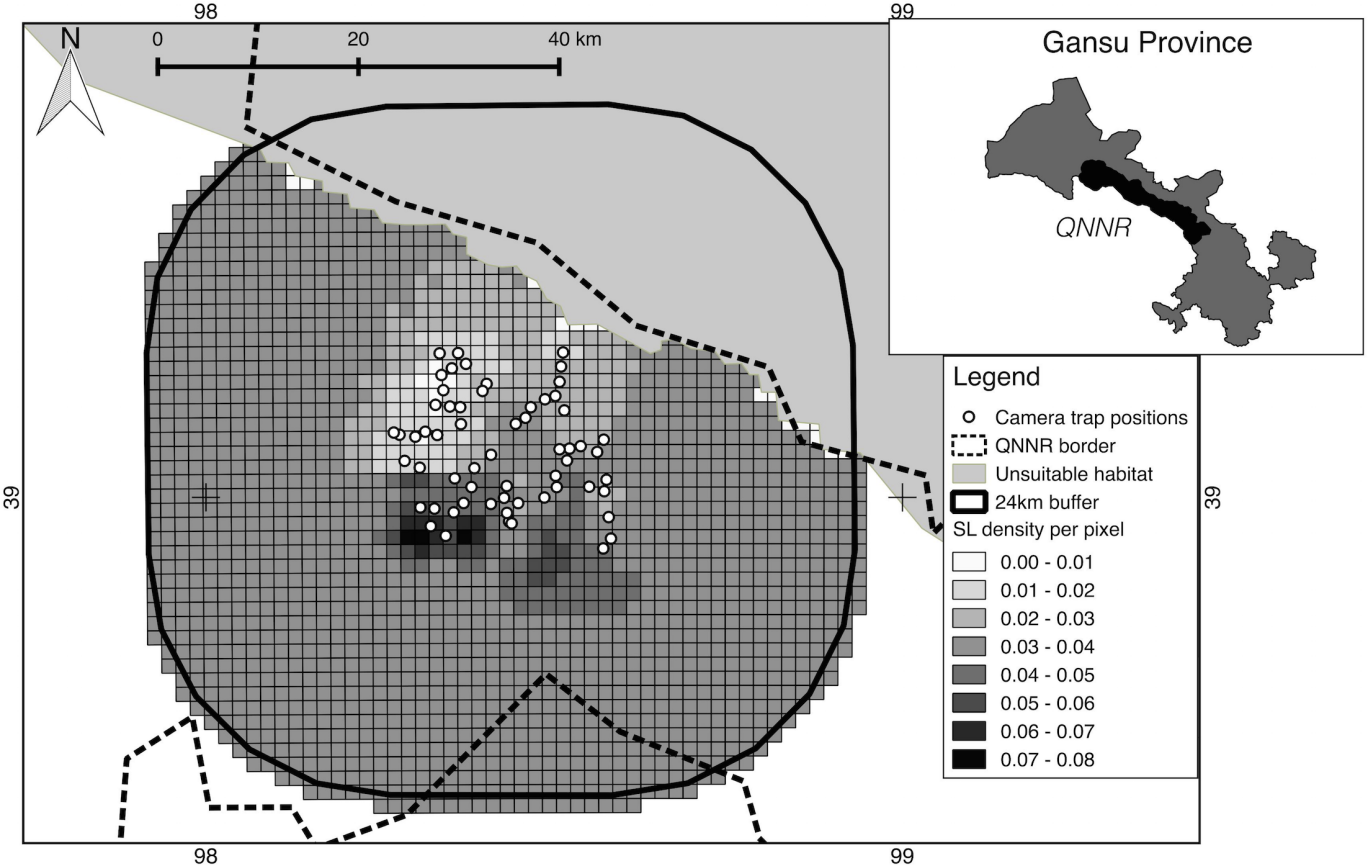
The map of the spatial distribution of snow leopards across the study area. A pixelated density map produced in SPACECAP showing estimated snow leopard densities per pixel of size 1.96 km^2^.

From the posterior density estimates for each pixel (1.96 km^2^), the density scale per pixel ranges from 0.028 to 0.077 (Fig 4). Higher density areas thus have a density 3 times higher than that of lower density areas. We note the spatial variation and the high snow leopard density estimated in the South-eastern area of the camera trap array, which is an area known to be more remote from human disturbance. Results of our simulation exercise indicate that our estimates from the SECR model were not within good bounds of accuracy and precision (Table 3). Our estimated RMSE for density was less or equal to 0.87 and psi parameters was less or equal to 0.12. We did however achieve a high coverage probability (>97%) indicating that the posterior distribution of the estimated parameters represented the true probability distribution well.

**Table 3:** Simulation results showing the bias and precision of the posterior mean, mode and median for the density and psi parameter. Root-mean-squared-error (RMSE) and % coverage rates for the 95% highest posterior density (HPD) intervals are reported.

	Initial estimates		Simulation Results						% Coverage		
Parameter	Mean	SD	Mean	RMSE	Mode	RMSE	Median	RMSE	Mean	Mode	Median
Density	3.31	1.01	3.48	0.84	3.27	0.87	3.41	0.84	97%	97%	97%
psi	0.50	0.15	0.51	0.11	0.48	0.12	0.50	0.11	99%	97%	97%

Covariates were not found to be strongly collinear (all Pearson correlation coefficients: less or equal to 0.40). The goodness-of-fit test for the Negative Binomial global model had a relatively good fit (residual deviance = 251, degrees of freedom = 248, p-value = 0.42). The Negative Binomial model estimated the over-dispersion parameter *θ* as 133.8 (SE 12.3), indicative of over-dispersion. The presence of the over-dispersion parameter indicated to us that we might have omitted some other important explanatory variables.

Across all seven Negative Binomial Model regressions, prey presence and grazing activity were the most influential covariate determining probability of snow leopard density within the sampled areas, both giving cumulative AIC weights of 0.99 (Table 4). Two models were considered adequate with a delta-AIC < 2. The most parsimonious model, which included prey presence and grazing activity as covariates, suggested a significant negative relationship between these covariates and snow leopard density (Table 5). The second best model included the effects of slope variation, although slope variation had no significant independent influence on snow leopard density.

**Table 4:** Negative Binomial Models quantifying the influence of factors on estimates of snow leopard abundance. Rankings are based on Akaike’s Information Criterion (AIC). Also includes relative parameter importance with summed AIC weights. (K = Number of parameters in the model; AIC wt = AIC model weight; AIC cum wt = AIC cumulative model weight).

							Parameter		
	Model	K	AIC	Delta AIC	AICwt	AIC cum. wt	Grazing	Prey	Slope
1	Abundance ~ Prey+ Grazing	4	3585.51	0	0.50	0.50	1	1	0
2	Abundance ~ Slope + Prey+ Grazing	5	3585.57	0.05	0.48	0.98	1	1	1
3	Abundance ~ Slope+ Prey	4	3592.78	7.27	0.01	0.99	0	1	1
4	Abundance ~ Grazing	3	3593.62	8.10	0.01	1.00	1	0	0
5	Abundance ~ Slope	3	3615.45	29.94	0.00	1.00	0	0	1
6	Abundance ~ Prey	3	3616.15	30.64	0.00	1.00	0	1	0
7	Abundance ~ Slope+ Grazing	4	3617.03	31.52	0.00	1.00	1	0	1
		Relative parameter importance (summed AIC wt)					0.99	0.99	0.50

**Table 5:** Negative Binomial top two models (delta-AIC < 2) quantifying the influence of covariates on estimates of snow leopard abundance.

	Model 1: Abundance ~ Prey+ Grazing			Model 2: Abundance ~ Prey+ Grazing+ Slope		
Parameter	Coefficient	SE	*P* value	Coefficient	SE	*P* value
Intercept	8.108	0.006	< 2e-16	8.108	0.006	< 2e-16
Prey	-0.019	0.001	0.001	-0.019	0.006	0.002
Grazing	-0.036	0.006	3.39e-09	-0.037	0.006	2.15e-09
Slope	-	-	-	-0.008	0.006	0.162

## Discussion

The assessment of snow leopard densities poses many practical challenges, due to the harsh conditions of the high-elevation regions they inhabit and their low-density dispersal over large home ranges [11]. To ensure sufficient sample sizes for density estimates, data collection needs to be carried out over large continuous areas. This was possible in QNNR during the winter when many areas are only accessible when water levels drop and rivers freeze. In such circumstances, assessments along the lines presented here have merits; they are simple and can be readily repeated. However, greater efficiency in data collection should be sought, in an effort to optimise the precision and reliability of density estimates.

We identified only a few studies that used CR methods for estimating snow leopard density. One was conducted in Xinjiang (estimated density 0.74/100 km^2^ [15]), and others in Ladakh, India (4.45–8.49/100 km^2^ individuals [11]), the Tien Shan Mountains of China and Kyrgyzstan (0.15–0.87/100 km^2^ [15]) and the Gobi Desert of Mongolia (0.70–1.50/100 km^2^ [27]). Our estimate of snow leopard density in QNNR at 3.31/100 km^2^ (SE = 1.01) falls within these ranges. Our estimate may be high, however, given that many snow leopards were only photographed once and we lack information on home range size and individual movements. Snow leopard densities are likely to vary across their range under the influence of key factors including habitat characteristics and prey density. The differences in density estimates between sites may therefore reflect true differences in abundance, or they may be due to the methods applied. For instance, our density estimates were more than doubled (to 8.31/100 km^2^) when we applied the non-spatial model, similar to the higher estimates from the Ladakh study that also used non-spatial models. There is a need for more systematic and coordinated research efforts using the SECR model to allow meaningful comparisons across the multi-country sites.

### Improving the performance of camera trapping

The SECR analysis reported here still suffers from low numbers of snow leopard captures and re-captures, with large standard error and high RMSE values. More effort needs to be invested in improving the quantity and quality of the data inputted into the model.

A promising technique for monitoring snow leopards is individual identification through DNA analysis of scats [15,54]. Major drawbacks to the use of DNA-based monitoring include the difficulty to obtain high quality and quantity samples of target DNA and the cost and scarcity of the necessary laboratory expertise [55]. Technological advances, including optimization of genetic techniques, may ease these constraints in the future. However at the present time camera traps provide a relatively low cost (depending on the models used) and user-friendly alternative that can be applied widely. While camera traps are often limited in the geographic area they cover, they represent an important tool that can readily used by local teams to appraise densities and threats and inform wider research.

In this study, we explored a camera trap set-up option where only one camera trap was deployed at each camera station [26], instead of the widely recommended two camera traps capturing images of both flanks of the animal for individualized identification [11,15]. Our approach aimed to optimise the use of scarce resources by doubling the survey area and thereby increasing the potential number of captures. Snow leopards were identified by the distinct pattern around the face, especially the forehead. The facial area has shorter fur and highly distinct areas and this technique is commonly used with snow leopards in captivity [56]. One drawback is that frontal images do not allow for sex identification [57], but this is problematic with photographic data of snow leopards in most circumstances.

When single cameras are used at each station it is even more important to optimize the quality of photographs for individual identification. In our case a significant proportion of photos were unusable for individual identification due to the poor quality of shots taken at night or of rapidly moving snow leopards. The likely benefits of investing in high quality cameras are substantial, although the financial implications may not be trivial for poorly resourced local teams. Careful consideration could also be given to using flash photography in order to improve night time photo quality and drawing the snow leopard’s interest in the camera by using attraction lures [58].

### Appraisal of threats

Threats are context-specific and we argue these should be carefully appraised at the local level. Livestock grazing is found throughout Central Asia’s high altitude snow leopard range and can lead to conservation challenges, for example, when overgrazing reduces the quantity and quality of rangeland resources for the principal prey species of the snow leopard [59–62]. We have documented the continued grazing of small stock and yak within the nature reserve, and observed that blue sheep were the most abundant wild ungulate. Other ungulates favoured by snow leopards, such as ibex *Capra sibirica* and Himalayan tahr *Hemitragus jemlahicus*, were not present [63].

We have demonstrated, however, that using available information on potential threats through GIS or sign-based surveys is not straightforward. Counter-intuitive covariate patterns (for example the negative effects of prey) were obtained across our set of models. The patterns observed should be interpreted with caution. The high amount of over-dispersion arising from our data may well mask the true effects of the covariates. When we utilized the Negative Binomial model to capture the over-dispersion we appeared to detect misleading “signals” amidst all the noise.

Possible explanations for over-dispersion here may relate to the covariate data used. For example, the scale and heterogeneity of covariates relative to snow leopard density estimates may be analytically incompatible. Sign-based indices that do not take into account detection probability [64] may lead to unreliable estimates of prey or grazing activity and unpredictable relationships with density. Our results identified substantial between-cell heterogeneity in covariate estimates, which highlight the need to develop further the approaches used and improve the measurement of potential determinants of snow leopards density. Given the analytical and logistical challenges we highlight, there is an urgent need to appraise threats through more robust quantitative methods, especially in the context of limited resources available for such work. Over-dispersion may also be the result of high estimation error in local snow leopard densities at the pixel level.

SECR models are rapidly being extended to allow for explicit inference about space usage, for example through the direct incorporation of covariates [65,66]. In this study, due to paucity of data, we did not explicitly include the covariates within the model, but explored what this analysis might imply. We also sought to combine multiple non-invasive techniques, including camera trapping and sign surveys, in order to provide a more multifaceted account of determinants of snow leopard spatial patterns. Our results raise caution regarding reliance on relatively coarse indexes for inferring relationships governing snow leopard density [67].

### SECR for snow leopards

As suggested above, further applications of SECR across the snow leopard range will allow for more formalized approaches in comparing density estimates across sites and over time. If repeated over multiple time periods, extensions of the CR framework will allow for additional applications, including the estimation of vital rates (survival, recruitment, movement) that drive changes in abundance [68].

There is further scope in at least four areas to expand the ambit of data collection and analysis. Firstly, we recommend the use of faecal DNA sampling during the process of camera trap data collection in order to further optimize field sampling procedures and increase precision in estimates of snow leopard density [69]. Secondly, additional behavioural data on snow leopards collected through other methods, such as the use of GPS collaring, can be integrated into the SECR models [65]. Finally, more comprehensive and fine-grained assessments of covariates of interest would be useful. When considering the influence of prey for example, more rigorous population density estimates for wild prey species and their spatial dynamics should be developed, using line transect distance sampling [6,70] or prey occupancy survey approaches [51,71]. Hence, we emphasize that our assessments of potential covariates is, at best, an exploratory analysis that shows promise for future work.

## Conclusion

The snow leopard is a crucial apex predator in high altitude mountain ecosystems and has the potential to act as an iconic barometer of the ecological condition of such settings. They are however exposed to many threats, including the growing impact of humans, who are increasingly encroaching on their habitat. Yet, surprisingly little is known about their ecology and distribution, especially within China [72]. This study sought to contribute to closing this knowledge gap, by using practical and systematic survey methods to estimate snow leopard numbers and identify the main threats that they face in a specific part of their range. Implementation was relatively simple, notwithstanding considerable logistical challenges. This approach enables the involvement of local teams, including protected area staff and citizen scientists, who can carry out these surveys rapidly and independently. For this purpose, performance improvements are necessary through measures to increase camera capture and photographic data quality. Concomitant improvements in approaches to assess prey abundance are also required to better determine relationships between prey and snow leopard abundance. We suggest that the described approach can be useful in rapid assessments of snow leopard density in other settings, in the context of wider monitoring efforts.

## Supporting Information

S1 Appendix Spatial kriging map of distribution probabilities for prey and grazing. We estimated and mapped probabilities of prey presence and livestock grazing activity for surveyed area within QNNR, Gansu Province. Kriging generates probabilities of prey or grazing presence in the landscape with white areas depicting low and black areas depicting high probability.
(DOCX)
