# Supplementary material for: Face Value: Towards Robust Estimates of Snow Leopard Densities
Source: PLoS One. 2015 Aug 31;10(8):e0134815. doi: 10.1371/journal.pone.0134815 (PMC4554729; doi:10.1371/journal.pone.0134815)
Supplement: S1 Appendix — We estimated and mapped probabilities of prey presence and livestock grazing activity for surveyed area within QNNR, Gansu Province. Kriging generates probabilities of prey or grazing presence in the landscape with white areas depicting low and black areas depicting high probability. (DOCX) [file pone.0134815.s001.docx]

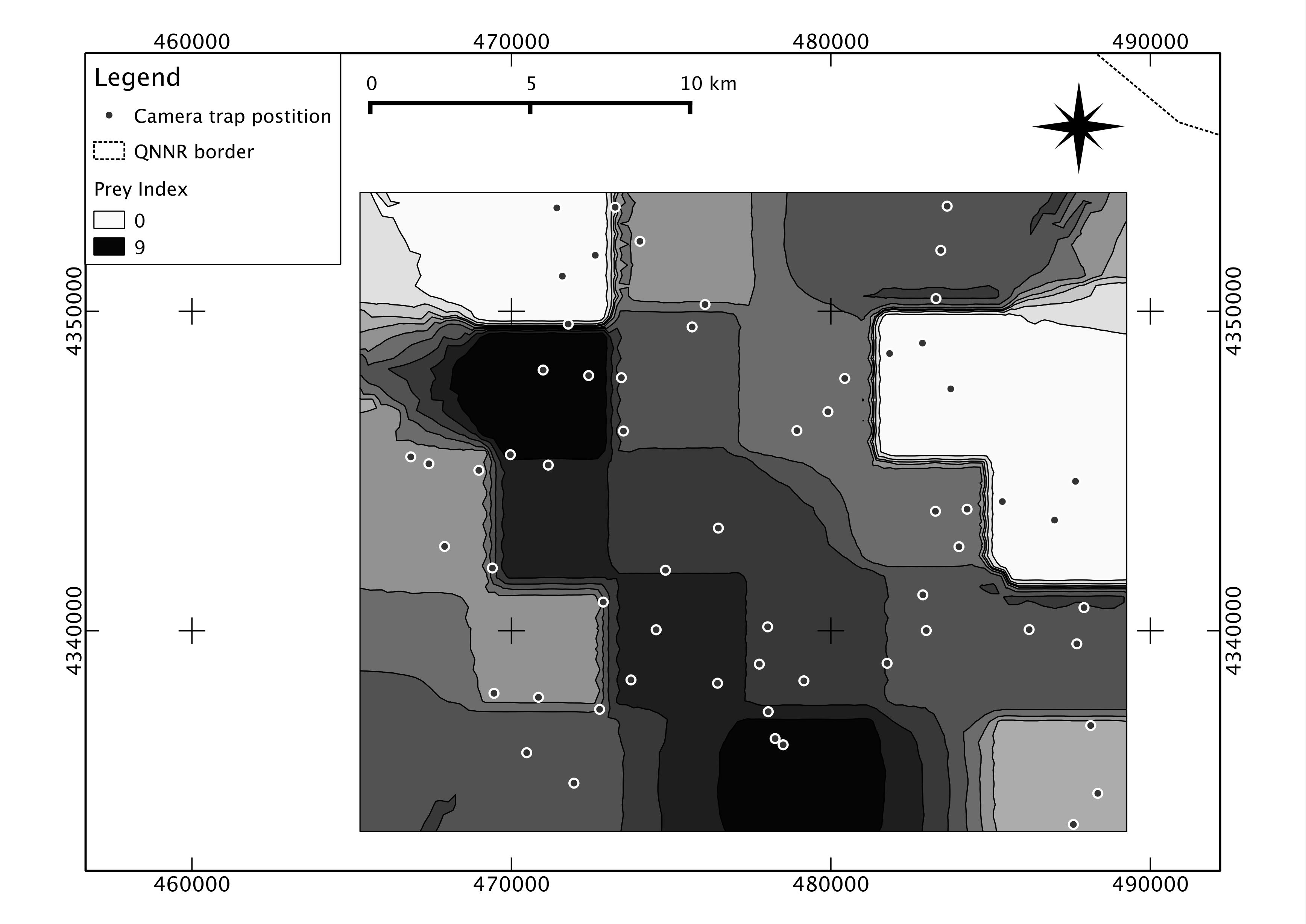


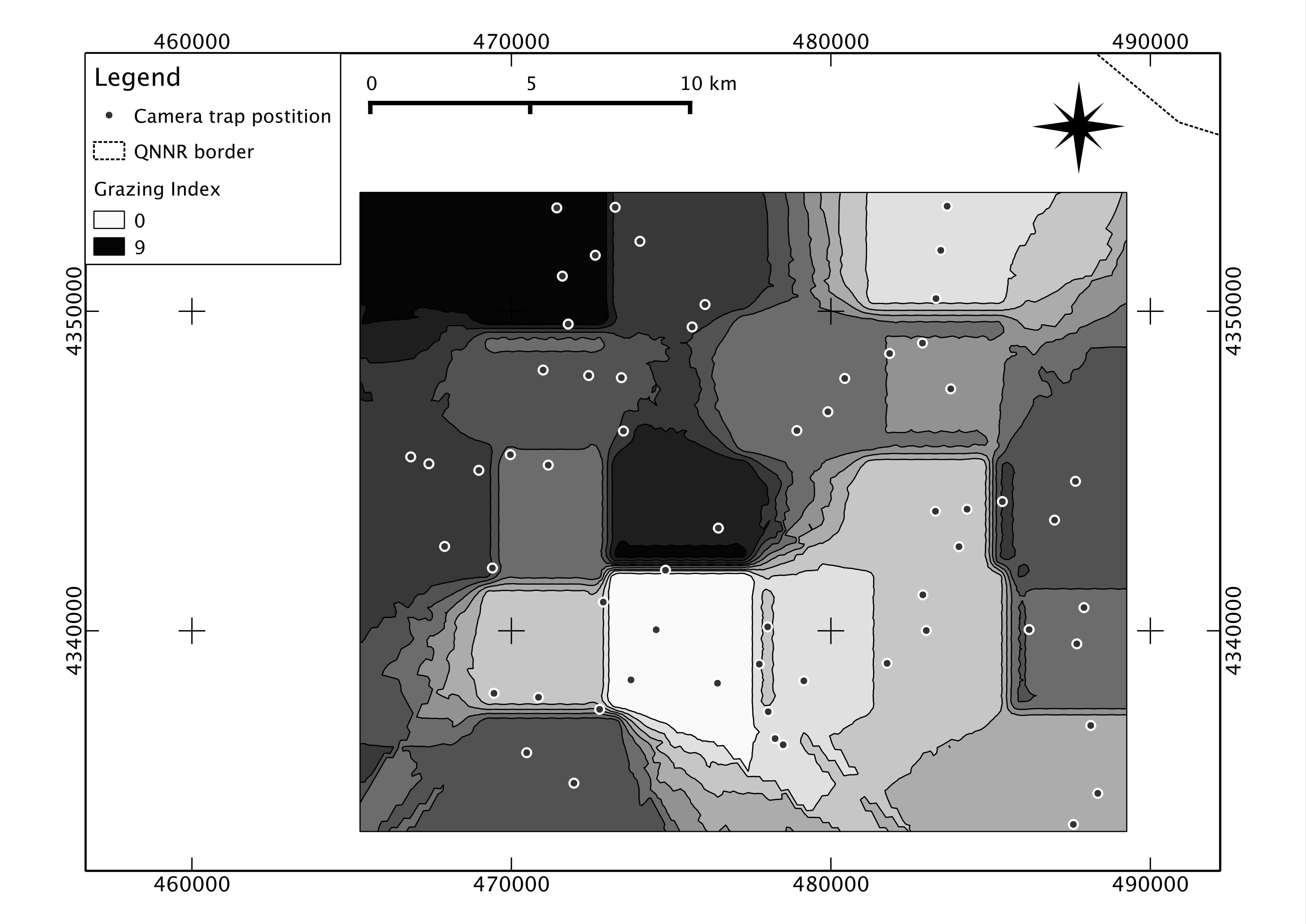


**S1 Appendix:** **Spatial kriging map of distribution probabilities for prey and grazing.** We estimated and mapped probabilities of prey presence and livestock grazing activity for surveyed area within QNNR, Gansu Province. Kriging generates probabilities of prey or grazing presence in the landscape with white areas depicting low and black areas depicting high probability.
